# Chlorido(chloro­diphenyl­phosphine-κ*P*)(diphenyl­piperidinophosphine-κ*P*)(η^5^-penta­methyl­cyclo­penta­dien­yl)ruthenium(II)

**DOI:** 10.1107/S1600536809027676

**Published:** 2009-07-18

**Authors:** Florian Jantscher, Karl Kirchner, Kurt Mereiter

**Affiliations:** a Institute of Applied Synthetic Chemistry, Vienna University of Technology, Getreidemarkt 9/163, A-1060 Vienna, Austria; b Institute of Chemical Technologies and Analytics, Vienna University of Technology, Getreidemarkt 9/164SC, A-1060 Vienna, Austria

## Abstract

The title compound, [Ru(C_10_H_15_)Cl(C_12_H_10_ClP)(C_17_H_20_NP)], is a half-sandwich complex of Ru^II^ with the chloro­diphenyl­phosphine ligand formed from the diphenyl­piperidinophosphine and chlorine of the precursor complex [Ru(η^5^-C_5_Me_5_)(κ^1^P—Ph_2_PNC_5_H_10_)Cl_2_] by an unexpected reaction with NaBH_4_. The complex has a three-legged piano-stool geometry, with Ru—P bond lengths of 2.2598 (5) Å for the chloro­phosphine and 2.3303 (5) Å for the amino­phosphine.

## Related literature

For general background to the reaction of half-sandwich ruthenium amino­phosphine complexes with diynes, see: Pavlik *et al.* (2006 ▶). For the unexpected formation and the crystal structure of a related Ru chloro­diphenyl­phosphine complex, see: Torres-Lubia *et al.* (1999 ▶). For the unexpected formation of another Mn chloro­diphenyl­phosphine complex, see: Liu *et al.* (1995 ▶). For the preparation of [Ru(Cp*)Cl_2_]_2_, see: Oshima *et al.* (1984 ▶).
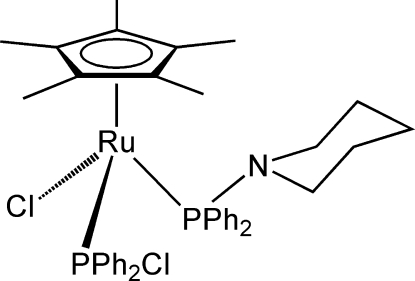


## Experimental

### 

#### Crystal data

[Ru(C_10_H_15_)Cl(C_12_H_10_ClP)(C_17_H_20_NP)]
*M* *_r_* = 761.67
Monoclinic, 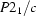

*a* = 17.5427 (12) Å
*b* = 9.1014 (6) Å
*c* = 22.3459 (15) Å
β = 98.881 (1)°
*V* = 3525.0 (4) Å^3^
*Z* = 4
Mo *K*α radiation
μ = 0.72 mm^−1^
*T* = 173 K
0.58 × 0.45 × 0.39 mm

#### Data collection

Bruker SMART APEX CCD diffractometer
Absorption correction: multi-scan (*SADABS*; Bruker, 2003 ▶) *T*_min_ = 0.61, *T*_max_ = 0.76
26236 measured reflections
10162 independent reflections
8628 reflections with *I* > 2σ(*I*)
*R*_int_ = 0.025

#### Refinement

*R*[*F* ^2^ > 2σ(*F* ^2^)] = 0.033
*wR*(*F* ^2^) = 0.083
*S* = 1.03
10162 reflections
411 parameters
H-atom parameters constrained
Δρ_max_ = 1.10 e Å^−3^
Δρ_min_ = −0.97 e Å^−3^

### 

Data collection: *SMART* (Bruker, 2003 ▶); cell refinement: *SAINT* (Bruker, 2003 ▶); data reduction: *SAINT*, *SADABS* and *XPREP* (Bruker, 2003 ▶); program(s) used to solve structure: *SHELXS97* (Sheldrick, 2008 ▶); program(s) used to refine structure: *SHELXL97* (Sheldrick, 2008 ▶); molecular graphics: *SHELXTL* (Sheldrick, 2008 ▶); software used to prepare material for publication: *SHELXTL*.

## Supplementary Material

Crystal structure: contains datablocks global, I. DOI: 10.1107/S1600536809027676/gk2223sup1.cif

Structure factors: contains datablocks I. DOI: 10.1107/S1600536809027676/gk2223Isup2.hkl

Additional supplementary materials:  crystallographic information; 3D view; checkCIF report

## Figures and Tables

**Table 1:** Selected bond lengths (Å)

Ru—C1	2.251 (2)
Ru—C2	2.274 (2)
Ru—C3	2.209 (2)
Ru—C4	2.240 (2)
Ru—C5	2.250 (2)
Ru—Cl1	2.4587 (5)

**Fig. 1.**
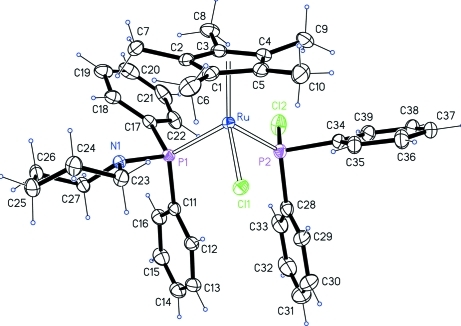
Perspective view of the title compound with the atom numbering scheme. Displacement ellipsoids are at the 30% probability level.

## supplementary crystallographic information

### Comment

We found that halfsandwich ruthenium aminophosphine complexes [Ru(Cp')(κ^1^*P*—Ph_2_PNRR')_2_(CH_3_CN)_2_]^+^ (Cp' is either Cp = cyclopentadienyl or Cp* = pentamethylcyclopentadienyl; Ph = phenyl; NRR' = NH*^n^*Pr, NEt_2_, or NC_5_H_10_ = piperidin-1-yl) react with diynes to afford novel η^3^-phosphaallyl-η^2^-vinylamine complexes and may transform under certain conditions to aminocarbenes (Pavlik *et al.*, 2006). In continuation of this work we were interested to react [Ru(Cp*)(κ^1^*P*—Ph_2_PNC_5_H_10_)Cl_2_], a Ru(III) complex, with NaBH_4_ in order to obtain a hydrido or borohydride complex. After workup of the reaction, NMR spectra indicated that another unknown Ru complex must have formed which was studied subsequently with X-ray diffraction and is reported here. The title compound, (I), turned out to contain, in addition to an intact piperidinodiphenylphosphine (Ph_2_PNC_5_H_10_), a chlorodiphenylphosphine as the second phosphine ligand of a half-sandwich complex with a three-legged piano-stool structure (Fig. 1). It is a Ru^II^ complex with the chemical formula [Ru(Cp*)(κ^1^*P*—Ph_2_PCl)(κ^1^*P*—Ph_2_PNC_5_H_10_)Cl] that bears similarities to [Ru(Cp*)(κ^1^*P*—Ph_2_PCl)_2_Cl] (Torres-Lubia *et al.*, 1999) with respect to stereochemistry of the complex, but also with respect to its formation. In case of [Ru(Cp*)(κ^1^*P*—Ph_2_PCl)_2_Cl] the chlorophosphine was not directly introduced but formed from [Ru(Cp*)(κ^1^*P*—Ph_2_PH)_2_Cl] (Ph_2_PH = hydridodiphenylphosphine) in CDCl_3_ as the solvent in the presence of the strong base DBN (1,5-diazabicyclo[4.3.0]non-5-ene) by a stepwise chlorine/hydride exchange between the solvent and the two hydridophosphines. In a related way the title compound must have formed from [Ru(Cp*)(κ^1^*P*—Ph_2_PNC_5_H_10_)Cl_2_] and NaBH_4_. However, there were no chlorinated solvents present in our reaction and the chlorine of the generated chlorophosphine must originate from the starting complex. It can be speculated that the formation of the title compound involves the intermediary formation of a hydridodiphenylphosphine, which by a hydride/chloride substitution leads to the chlorodiphenylphosphine of the title compound obtained in a poor yield of only 9%. A related and also unexpected transformation of a thiol-substituted hydridodiphenylphosphine into a thiol-substituted chlorodiphenylphosphine was observed in the reaction with Mn(CO)_5_Br in chloroform (Liu *et al.*, 1995). Bond lengths and angles in the title compound (see Table 1 and supplementary materials) are similar to those of [Ru(Cp*)(κ^1^*P*—Ph_2_PCl)_2_Cl] (Torres-Lubia *et al.*, 1999), which has <Ru—C> = 2.246 (20) Å (2.245 (21) Å for (I)), Ru—P = 2.242 (2) and 2.257 (2) Å, Ru—Cl = 2.438 (2) Å, and P—Cl = 2.085 (3) Å. Both Ru complexes adopt related conformations and have in common that the Ru-bonded and the P-bonded Cl lie on opposite sides. In both complexes the phosphine ligands adopt orientations that lead to a stabilization by intramolecular π-π-stacking between the two adjacent phenyl rings (Fig. 1) with a shortest contact distance of C(12)···C(28) = 3.183 (3) Å in the title compound (ring-ring centroid distance 3.717 (2) Å, ring-ring inclination angle 12.96°). Moreover, it is remarkable that the title compound shows eight intramolecular C—H···Cl/N interactions (seven to Cl, one to N), but only one intermolecular C—H···Cl interaction (see supplementary materials).

### Experimental

The synthesis of [Ru(Cp*)(κ^1^*P*-PPh_2_NC_5_H_10_)(κ^1^*P*-PPh_2_Cl)Cl], (I), was carried out as follows: A solution of [Ru(Cp*)Cl_2_]_2_ (322 mg, 0.52 mmol) (Oshima *et al.*, 1984) in THF (10 ml) in THF (10 ml) was treated with Ph_2_PNC_5_H_10_ (300 mg, 1.2 mmol) and the solution was stirred for 1 h at room temperature. After that NaBH_4_ (150 mg, 4.0 mmol) was added and the mixture was stirred for 12 h. After evaporation of the solvent an oily residue was obtained from which the product was extracted with pentane (2 *x* 10 ml). The volume of the solution was then reduced to about 0.5 ml whereupon red crystals of the title compound were obtained. Yield: 42 mg (9%). ^1^H NMR (C_6_D_6_, 293 K, δ, p.p.m.): 7.50 - 6.51 [m, 20H, Ph], 3.03 – 2.72 [m, 4H, CH_2_], 1.40 [s, 15H, Cp*], 1.33 – 1.26 [m, 4H, CH_2_], 1.00 – 0.89 [m, 2H, CH_2_]. ^13^C{^1^H} NMR (C_6_D_6_, 293 K, δ, p.p.m.): 137.1 – 125.7 [Ph], 93.2 [Cp*], 45.86 [CH_2_], 26.2 [CH_2_], 24.6 [CH_2_], 8.9 [Cp*]. ^31^P{^1^H} NMR (acetone-d_6_, 293 K, δ, p.p.m.): 93.1 [d, J_HP_ = 37.2 Hz, PPh_2_Cl], 36.0 [d, J_HP_ = 38.5 Hz, PPh_2_N]. Crystals for X-ray diffraction were obtained by cooling a pentane solution to -20°C.

### Refinement

All H atoms were placed in calculated positions, with C—H = 0.93–0.98 Å, and were thereafter treated as riding, with *U*_iso_(H) values of 1.5*U*_eq_(C) for methyl groups and 1.2*U*_eq_(C) for others.

### Crystal data

**Table:**

[Ru(C_10_H_15_)Cl(C_12_H_10_ClP)(C_17_H_20_NP)]	*F*(000) = 1576
*M**_r_* = 761.67	*D*_x_ = 1.435 Mg m^−^^3^
Monoclinic, *P*2_1_/*c*	Mo *K*α radiation, λ = 0.71073 Å
Hall symbol: -P 2ybc	Cell parameters from 8233 reflections
*a* = 17.5427 (12) Å	θ = 2.3–30.0°
*b* = 9.1014 (6) Å	µ = 0.72 mm^−^^1^
*c* = 22.3459 (15) Å	*T* = 173 K
β = 98.881 (1)°	Block, orange
*V* = 3525.0 (4) Å^3^	0.58 × 0.45 × 0.39 mm
*Z* = 4	

### Data collection

**Table:**

Bruker SMART APEX CCD diffractometer	10162 independent reflections
Radiation source: fine-focus sealed tube	8628 reflections with *I* > 2σ(*I*)
graphite	*R*_int_ = 0.025
ω scans	θ_max_ = 30.0°, θ_min_ = 2.4°
Absorption correction: multi-scan (*SADABS*; Bruker, 2003)	*h* = −24→20
*T*_min_ = 0.61, *T*_max_ = 0.76	*k* = −12→11
26236 measured reflections	*l* = −30→31

### Refinement

**Table:**

Refinement on *F*^2^	Primary atom site location: structure-invariant direct methods
Least-squares matrix: full	Secondary atom site location: difference Fourier map
*R*[*F*^2^ > 2σ(*F*^2^)] = 0.033	Hydrogen site location: inferred from neighbouring sites
*wR*(*F*^2^) = 0.083	H-atom parameters constrained
*S* = 1.03	*w* = 1/[σ^2^(*F*_o_^2^) + (0.0326*P*)^2^ + 2.7631*P*] where *P* = (*F*_o_^2^ + 2*F*_c_^2^)/3
10162 reflections	(Δ/σ)_max_ = 0.001
411 parameters	Δρ_max_ = 1.10 e Å^−^^3^
0 restraints	Δρ_min_ = −0.97 e Å^−^^3^

### Special details

**Table:**

Geometry. All e.s.d.'s (except the e.s.d. in the dihedral angle between two l.s. planes) are estimated using the full covariance matrix. The cell e.s.d.'s are taken into account individually in the estimation of e.s.d.'s in distances, angles and torsion angles; correlations between e.s.d.'s in cell parameters are only used when they are defined by crystal symmetry. An approximate (isotropic) treatment of cell e.s.d.'s is used for estimating e.s.d.'s involving l.s. planes.
Refinement. Refinement of *F*^2^ against ALL reflections. The weighted *R*-factor *wR* and goodness of fit *S* are based on *F*^2^, conventional *R*-factors *R* are based on *F*, with *F* set to zero for negative *F*^2^. The threshold expression of *F*^2^ > σ(*F*^2^) is used only for calculating *R*-factors(gt) *etc*. and is not relevant to the choice of reflections for refinement. *R*-factors based on *F*^2^ are statistically about twice as large as those based on *F*, and *R*- factors based on ALL data will be even larger.

### Fractional atomic coordinates and isotropic or equivalent isotropic displacement parameters (Å^2^)

**Table:**

	*x*	*y*	*z*	*U*_iso_*/*U*_eq_	
Ru	0.319239 (8)	0.284822 (17)	0.404551 (6)	0.02488 (4)	
Cl1	0.35958 (3)	0.03019 (6)	0.39015 (2)	0.03404 (10)	
Cl2	0.22523 (4)	0.50673 (8)	0.27604 (3)	0.05243 (16)	
P1	0.19442 (3)	0.22618 (5)	0.41995 (2)	0.02501 (9)	
P2	0.29029 (3)	0.31711 (6)	0.30332 (2)	0.03087 (11)	
N1	0.18748 (9)	0.12798 (18)	0.48395 (7)	0.0268 (3)	
C1	0.40816 (12)	0.2978 (2)	0.48857 (9)	0.0345 (4)	
C2	0.34730 (11)	0.3924 (2)	0.49720 (9)	0.0323 (4)	
C3	0.33811 (12)	0.5006 (2)	0.44971 (10)	0.0355 (4)	
C4	0.39950 (13)	0.4777 (3)	0.41486 (10)	0.0388 (5)	
C5	0.44181 (11)	0.3528 (3)	0.43769 (10)	0.0372 (4)	
C6	0.43962 (14)	0.1718 (3)	0.52760 (11)	0.0486 (6)	
H6A	0.4953	0.1848	0.5399	0.073*	
H6B	0.4300	0.0799	0.5048	0.073*	
H6C	0.4143	0.1681	0.5637	0.073*	
C7	0.30928 (13)	0.3945 (3)	0.55296 (10)	0.0412 (5)	
H7A	0.3482	0.4133	0.5886	0.062*	
H7B	0.2847	0.2993	0.5575	0.062*	
H7C	0.2701	0.4722	0.5492	0.062*	
C8	0.28894 (15)	0.6353 (3)	0.44579 (13)	0.0481 (6)	
H8A	0.3204	0.7195	0.4620	0.072*	
H8B	0.2469	0.6208	0.4695	0.072*	
H8C	0.2672	0.6539	0.4034	0.072*	
C9	0.42049 (18)	0.5852 (3)	0.36886 (12)	0.0565 (7)	
H9A	0.4466	0.6702	0.3898	0.085*	
H9B	0.3735	0.6179	0.3427	0.085*	
H9C	0.4550	0.5375	0.3442	0.085*	
C10	0.51677 (13)	0.2956 (4)	0.42237 (13)	0.0552 (7)	
H10A	0.5587	0.3198	0.4552	0.083*	
H10B	0.5270	0.3410	0.3846	0.083*	
H10C	0.5135	0.1887	0.4173	0.083*	
C11	0.12841 (11)	0.1238 (2)	0.36245 (8)	0.0307 (4)	
C12	0.15773 (12)	−0.0043 (2)	0.34017 (9)	0.0346 (4)	
H12	0.2105	−0.0293	0.3520	0.041*	
C13	0.10999 (14)	−0.0957 (3)	0.30068 (10)	0.0430 (5)	
H13	0.1305	−0.1823	0.2855	0.052*	
C14	0.03307 (15)	−0.0612 (3)	0.28344 (11)	0.0491 (6)	
H14	0.0009	−0.1233	0.2562	0.059*	
C15	0.00306 (14)	0.0643 (3)	0.30598 (11)	0.0486 (6)	
H15	−0.0500	0.0878	0.2945	0.058*	
C16	0.05041 (12)	0.1562 (3)	0.34542 (10)	0.0400 (5)	
H16	0.0293	0.2418	0.3609	0.048*	
C17	0.13699 (10)	0.3886 (2)	0.43291 (9)	0.0309 (4)	
C18	0.12363 (11)	0.4305 (2)	0.49036 (11)	0.0365 (4)	
H18	0.1408	0.3696	0.5243	0.044*	
C19	0.08509 (13)	0.5620 (3)	0.49828 (13)	0.0479 (6)	
H19	0.0764	0.5900	0.5376	0.057*	
C20	0.05979 (14)	0.6505 (3)	0.44984 (16)	0.0576 (7)	
H20	0.0343	0.7403	0.4556	0.069*	
C21	0.07135 (14)	0.6091 (3)	0.39243 (15)	0.0560 (7)	
H21	0.0530	0.6697	0.3587	0.067*	
C22	0.10972 (13)	0.4791 (3)	0.38403 (12)	0.0437 (5)	
H22	0.1175	0.4516	0.3444	0.052*	
C23	0.24435 (13)	0.0107 (2)	0.50082 (9)	0.0353 (4)	
H23A	0.2957	0.0434	0.4929	0.042*	
H23B	0.2295	−0.0776	0.4759	0.042*	
C24	0.24849 (14)	−0.0274 (3)	0.56766 (10)	0.0423 (5)	
H24A	0.2678	0.0588	0.5925	0.051*	
H24B	0.2854	−0.1090	0.5780	0.051*	
C25	0.16975 (15)	−0.0720 (3)	0.58253 (10)	0.0454 (5)	
H25A	0.1540	−0.1671	0.5629	0.055*	
H25B	0.1729	−0.0845	0.6269	0.055*	
C26	0.11003 (13)	0.0447 (3)	0.56040 (10)	0.0424 (5)	
H26A	0.1214	0.1352	0.5847	0.051*	
H26B	0.0582	0.0095	0.5661	0.051*	
C27	0.11023 (11)	0.0792 (2)	0.49367 (9)	0.0342 (4)	
H27A	0.0952	−0.0095	0.4690	0.041*	
H27B	0.0721	0.1574	0.4805	0.041*	
C28	0.24045 (12)	0.1834 (3)	0.24860 (9)	0.0390 (5)	
C29	0.28044 (15)	0.0544 (3)	0.24101 (11)	0.0486 (6)	
H29	0.3299	0.0391	0.2642	0.058*	
C30	0.24893 (19)	−0.0529 (4)	0.19986 (13)	0.0633 (8)	
H30	0.2767	−0.1410	0.1956	0.076*	
C31	0.1786 (2)	−0.0315 (4)	0.16586 (13)	0.0688 (9)	
H31	0.1574	−0.1042	0.1376	0.083*	
C32	0.13863 (18)	0.0936 (4)	0.17229 (12)	0.0656 (9)	
H32	0.0895	0.1075	0.1484	0.079*	
C33	0.16873 (15)	0.2039 (3)	0.21393 (11)	0.0517 (6)	
H33	0.1401	0.2909	0.2181	0.062*	
C34	0.37129 (12)	0.3511 (2)	0.26195 (9)	0.0340 (4)	
C35	0.43729 (13)	0.2691 (3)	0.27841 (10)	0.0406 (5)	
H35	0.4403	0.2018	0.3112	0.049*	
C36	0.49969 (15)	0.2849 (3)	0.24692 (12)	0.0476 (5)	
H36	0.5451	0.2283	0.2584	0.057*	
C37	0.49574 (15)	0.3824 (3)	0.19928 (11)	0.0476 (6)	
H37	0.5386	0.3946	0.1785	0.057*	
C38	0.42924 (15)	0.4617 (3)	0.18205 (10)	0.0473 (6)	
H38	0.4262	0.5274	0.1487	0.057*	
C39	0.36657 (14)	0.4471 (3)	0.21260 (9)	0.0414 (5)	
H39	0.3208	0.5020	0.2001	0.050*	

### Atomic displacement parameters (Å^2^)

**Table:**

	*U*^11^	*U*^22^	*U*^33^	*U*^12^	*U*^13^	*U*^23^
Ru	0.02481 (7)	0.02548 (8)	0.02439 (7)	−0.00146 (5)	0.00396 (5)	−0.00187 (5)
Cl1	0.0356 (2)	0.0312 (2)	0.0362 (2)	0.00403 (18)	0.00831 (18)	−0.00388 (18)
Cl2	0.0585 (3)	0.0544 (4)	0.0469 (3)	0.0194 (3)	0.0158 (3)	0.0190 (3)
P1	0.0252 (2)	0.0242 (2)	0.0253 (2)	−0.00082 (16)	0.00303 (16)	0.00145 (17)
P2	0.0303 (2)	0.0367 (3)	0.0258 (2)	−0.00082 (19)	0.00501 (17)	0.00290 (19)
N1	0.0287 (7)	0.0258 (8)	0.0260 (7)	−0.0005 (6)	0.0047 (6)	0.0015 (6)
C1	0.0316 (9)	0.0404 (11)	0.0295 (9)	−0.0002 (8)	−0.0017 (7)	−0.0072 (8)
C2	0.0314 (9)	0.0342 (10)	0.0304 (9)	−0.0053 (7)	0.0022 (7)	−0.0088 (8)
C3	0.0364 (10)	0.0293 (10)	0.0402 (10)	−0.0059 (8)	0.0045 (8)	−0.0070 (8)
C4	0.0398 (11)	0.0393 (12)	0.0377 (10)	−0.0159 (9)	0.0072 (8)	−0.0078 (9)
C5	0.0280 (9)	0.0460 (12)	0.0374 (10)	−0.0074 (8)	0.0039 (7)	−0.0114 (9)
C6	0.0464 (13)	0.0562 (15)	0.0382 (11)	0.0100 (11)	−0.0093 (9)	−0.0013 (11)
C7	0.0425 (11)	0.0486 (13)	0.0334 (10)	−0.0063 (10)	0.0088 (8)	−0.0122 (9)
C8	0.0512 (13)	0.0282 (11)	0.0642 (15)	−0.0027 (9)	0.0063 (11)	−0.0068 (10)
C9	0.0725 (18)	0.0477 (15)	0.0513 (14)	−0.0288 (13)	0.0160 (13)	−0.0018 (12)
C10	0.0295 (10)	0.082 (2)	0.0551 (14)	−0.0038 (11)	0.0088 (10)	−0.0193 (14)
C11	0.0322 (9)	0.0349 (10)	0.0242 (8)	−0.0063 (7)	0.0022 (7)	0.0045 (7)
C12	0.0393 (10)	0.0334 (10)	0.0304 (9)	−0.0065 (8)	0.0035 (7)	0.0017 (8)
C13	0.0545 (13)	0.0390 (12)	0.0350 (10)	−0.0149 (10)	0.0053 (9)	−0.0035 (9)
C14	0.0535 (14)	0.0559 (16)	0.0351 (11)	−0.0231 (12)	−0.0019 (10)	−0.0003 (10)
C15	0.0356 (11)	0.0644 (17)	0.0415 (12)	−0.0130 (11)	−0.0074 (9)	0.0077 (11)
C16	0.0335 (10)	0.0469 (13)	0.0380 (10)	−0.0047 (9)	0.0001 (8)	0.0036 (9)
C17	0.0249 (8)	0.0253 (9)	0.0429 (10)	0.0012 (7)	0.0065 (7)	0.0039 (8)
C18	0.0305 (9)	0.0307 (10)	0.0481 (11)	0.0006 (8)	0.0052 (8)	−0.0040 (9)
C19	0.0386 (11)	0.0355 (12)	0.0700 (16)	0.0014 (9)	0.0102 (11)	−0.0131 (11)
C20	0.0391 (12)	0.0288 (12)	0.106 (2)	0.0066 (9)	0.0150 (13)	0.0010 (13)
C21	0.0372 (12)	0.0409 (13)	0.091 (2)	0.0074 (10)	0.0150 (12)	0.0307 (14)
C22	0.0352 (10)	0.0412 (12)	0.0561 (13)	0.0039 (9)	0.0113 (9)	0.0173 (10)
C23	0.0408 (10)	0.0319 (10)	0.0337 (9)	0.0073 (8)	0.0079 (8)	0.0052 (8)
C24	0.0481 (12)	0.0440 (13)	0.0336 (10)	0.0051 (10)	0.0023 (9)	0.0113 (9)
C25	0.0590 (14)	0.0430 (13)	0.0344 (10)	−0.0071 (11)	0.0076 (10)	0.0104 (9)
C26	0.0444 (12)	0.0479 (13)	0.0372 (11)	−0.0064 (10)	0.0134 (9)	0.0075 (10)
C27	0.0329 (9)	0.0354 (11)	0.0346 (9)	−0.0050 (8)	0.0067 (7)	0.0047 (8)
C28	0.0355 (10)	0.0565 (14)	0.0246 (9)	−0.0111 (9)	0.0031 (7)	0.0030 (9)
C29	0.0499 (13)	0.0570 (16)	0.0392 (11)	−0.0112 (11)	0.0078 (10)	−0.0129 (11)
C30	0.0765 (19)	0.068 (2)	0.0471 (14)	−0.0179 (16)	0.0154 (13)	−0.0225 (14)
C31	0.083 (2)	0.080 (2)	0.0416 (14)	−0.0289 (19)	0.0032 (14)	−0.0154 (15)
C32	0.0586 (16)	0.094 (3)	0.0387 (13)	−0.0312 (17)	−0.0110 (11)	0.0103 (14)
C33	0.0443 (13)	0.0681 (18)	0.0399 (12)	−0.0131 (12)	−0.0024 (10)	0.0119 (12)
C34	0.0383 (10)	0.0373 (11)	0.0275 (9)	−0.0066 (8)	0.0083 (7)	−0.0019 (8)
C35	0.0414 (11)	0.0438 (12)	0.0393 (11)	−0.0010 (9)	0.0148 (9)	0.0013 (9)
C36	0.0448 (12)	0.0498 (14)	0.0529 (13)	−0.0012 (10)	0.0223 (10)	0.0002 (11)
C37	0.0573 (14)	0.0478 (14)	0.0438 (12)	−0.0160 (11)	0.0269 (11)	−0.0087 (10)
C38	0.0600 (15)	0.0535 (15)	0.0311 (10)	−0.0140 (12)	0.0157 (10)	−0.0004 (10)
C39	0.0492 (12)	0.0479 (13)	0.0270 (9)	−0.0081 (10)	0.0054 (8)	0.0027 (9)

### Geometric parameters (Å, °)

**Table:**

Ru—C1	2.251 (2)	C15—H15	0.9500
Ru—C2	2.274 (2)	C16—H16	0.9500
Ru—C3	2.209 (2)	C17—C22	1.393 (3)
Ru—C4	2.240 (2)	C17—C18	1.393 (3)
Ru—C5	2.250 (2)	C18—C19	1.399 (3)
Ru—P1	2.3303 (5)	C18—H18	0.9500
Ru—P2	2.2598 (5)	C19—C20	1.367 (4)
Ru—Cl1	2.4587 (5)	C19—H19	0.9500
P1—N1	1.7067 (16)	C20—C21	1.382 (4)
P1—C11	1.844 (2)	C20—H20	0.9500
P1—C17	1.837 (2)	C21—C22	1.388 (4)
P2—Cl2	2.1066 (8)	C21—H21	0.9500
P2—C28	1.846 (2)	C22—H22	0.9500
P2—C34	1.837 (2)	C23—C24	1.524 (3)
N1—C23	1.470 (2)	C23—H23A	0.9900
N1—C27	1.474 (2)	C23—H23B	0.9900
C1—C2	1.408 (3)	C24—C25	1.525 (3)
C1—C5	1.449 (3)	C24—H24A	0.9900
C1—C6	1.494 (3)	C24—H24B	0.9900
C2—C3	1.438 (3)	C25—C26	1.520 (4)
C2—C7	1.501 (3)	C25—H25A	0.9900
C3—C4	1.438 (3)	C25—H25B	0.9900
C3—C8	1.494 (3)	C26—C27	1.524 (3)
C4—C5	1.409 (3)	C26—H26A	0.9900
C4—C9	1.506 (3)	C26—H26B	0.9900
C5—C10	1.502 (3)	C27—H27A	0.9900
C6—H6A	0.9800	C27—H27B	0.9900
C6—H6B	0.9800	C28—C33	1.385 (3)
C6—H6C	0.9800	C28—C29	1.392 (4)
C7—H7A	0.9800	C29—C30	1.396 (4)
C7—H7B	0.9800	C29—H29	0.9500
C7—H7C	0.9800	C30—C31	1.359 (5)
C8—H8A	0.9800	C30—H30	0.9500
C8—H8B	0.9800	C31—C32	1.357 (5)
C8—H8C	0.9800	C31—H31	0.9500
C9—H9A	0.9800	C32—C33	1.414 (4)
C9—H9B	0.9800	C32—H32	0.9500
C9—H9C	0.9800	C33—H33	0.9500
C10—H10A	0.9800	C34—C35	1.379 (3)
C10—H10B	0.9800	C34—C39	1.400 (3)
C10—H10C	0.9800	C35—C36	1.397 (3)
C11—C16	1.394 (3)	C35—H35	0.9500
C11—C12	1.397 (3)	C36—C37	1.379 (4)
C12—C13	1.394 (3)	C36—H36	0.9500
C12—H12	0.9500	C37—C38	1.375 (4)
C13—C14	1.381 (4)	C37—H37	0.9500
C13—H13	0.9500	C38—C39	1.387 (3)
C14—C15	1.385 (4)	C38—H38	0.9500
C14—H14	0.9500	C39—H39	0.9500
C15—C16	1.393 (3)		
			
P1—Ru—P2	96.11 (2)	C16—C11—P1	124.58 (17)
P1—Ru—Cl1	95.71 (2)	C12—C11—P1	116.36 (15)
P2—Ru—Cl1	90.94 (2)	C13—C12—C11	120.3 (2)
N1—P1—C11	101.58 (8)	C13—C12—H12	119.8
N1—P1—C17	100.22 (8)	C11—C12—H12	119.8
C11—P1—C17	102.46 (9)	C14—C13—C12	120.4 (2)
Cl2—P2—C28	99.68 (8)	C14—C13—H13	119.8
Cl2—P2—C34	98.23 (8)	C12—C13—H13	119.8
C28—P2—C34	95.83 (9)	C13—C14—C15	119.7 (2)
C3—Ru—C4	37.71 (8)	C13—C14—H14	120.1
C3—Ru—C5	62.42 (8)	C15—C14—H14	120.1
C4—Ru—C5	36.58 (9)	C14—C15—C16	120.1 (2)
C3—Ru—C1	62.41 (8)	C14—C15—H15	119.9
C4—Ru—C1	61.93 (8)	C16—C15—H15	119.9
C5—Ru—C1	37.56 (8)	C15—C16—C11	120.7 (2)
C3—Ru—P2	109.66 (6)	C15—C16—H16	119.7
C4—Ru—P2	92.38 (6)	C11—C16—H16	119.7
C5—Ru—P2	110.44 (6)	C22—C17—C18	118.3 (2)
C1—Ru—P2	147.97 (6)	C22—C17—P1	118.87 (17)
C3—Ru—C2	37.39 (8)	C18—C17—P1	122.69 (16)
C4—Ru—C2	61.63 (8)	C17—C18—C19	120.3 (2)
C5—Ru—C2	61.35 (7)	C17—C18—H18	119.9
C1—Ru—C2	36.24 (7)	C19—C18—H18	119.9
P2—Ru—C2	147.03 (6)	C20—C19—C18	120.5 (3)
C3—Ru—P1	102.36 (6)	C20—C19—H19	119.7
C4—Ru—P1	138.97 (6)	C18—C19—H19	119.7
C5—Ru—P1	152.42 (5)	C19—C20—C21	119.9 (2)
C1—Ru—P1	115.77 (6)	C19—C20—H20	120.1
C2—Ru—P1	92.32 (5)	C21—C20—H20	120.1
C3—Ru—Cl1	150.65 (6)	C20—C21—C22	120.1 (2)
C4—Ru—Cl1	124.27 (6)	C20—C21—H21	119.9
C5—Ru—Cl1	91.30 (6)	C22—C21—H21	119.9
C1—Ru—Cl1	88.98 (6)	C21—C22—C17	120.9 (2)
C2—Ru—Cl1	119.91 (6)	C21—C22—H22	119.6
N1—P1—Ru	115.71 (6)	C17—C22—H22	119.6
C17—P1—Ru	112.94 (6)	N1—C23—C24	110.12 (17)
C11—P1—Ru	121.08 (6)	N1—C23—H23A	109.6
C34—P2—Ru	116.99 (7)	C24—C23—H23A	109.6
C28—P2—Ru	125.98 (7)	N1—C23—H23B	109.6
Cl2—P2—Ru	115.10 (3)	C24—C23—H23B	109.6
C2—C1—C5	107.78 (19)	H23A—C23—H23B	108.1
C2—C1—C6	127.5 (2)	C23—C24—C25	111.50 (19)
C5—C1—C6	124.4 (2)	C23—C24—H24A	109.3
C2—C1—Ru	72.77 (11)	C25—C24—H24A	109.3
C5—C1—Ru	71.18 (11)	C23—C24—H24B	109.3
C6—C1—Ru	126.36 (16)	C25—C24—H24B	109.3
C1—C2—C3	108.58 (18)	H24A—C24—H24B	108.0
C1—C2—C7	124.4 (2)	C26—C25—C24	110.18 (19)
C3—C2—C7	126.2 (2)	C26—C25—H25A	109.6
C1—C2—Ru	70.99 (11)	C24—C25—H25A	109.6
C3—C2—Ru	68.85 (11)	C26—C25—H25B	109.6
C7—C2—Ru	134.38 (14)	C24—C25—H25B	109.6
C4—C3—C2	107.00 (19)	H25A—C25—H25B	108.1
C4—C3—C8	124.1 (2)	C25—C26—C27	110.92 (19)
C2—C3—C8	127.1 (2)	C25—C26—H26A	109.5
C4—C3—Ru	72.30 (12)	C27—C26—H26A	109.5
C2—C3—Ru	73.76 (11)	C25—C26—H26B	109.5
C8—C3—Ru	131.17 (16)	C27—C26—H26B	109.5
C5—C4—C3	108.50 (19)	H26A—C26—H26B	108.0
C5—C4—C9	126.9 (2)	N1—C27—C26	110.32 (17)
C3—C4—C9	123.9 (2)	N1—C27—H27A	109.6
C5—C4—Ru	72.12 (12)	C26—C27—H27A	109.6
C3—C4—Ru	69.99 (11)	N1—C27—H27B	109.6
C9—C4—Ru	130.94 (16)	C26—C27—H27B	109.6
C4—C5—C1	107.90 (18)	H27A—C27—H27B	108.1
C4—C5—C10	128.9 (2)	C33—C28—C29	118.5 (2)
C1—C5—C10	122.4 (2)	C33—C28—P2	125.3 (2)
C4—C5—Ru	71.31 (11)	C29—C28—P2	116.14 (17)
C1—C5—Ru	71.26 (11)	C28—C29—C30	121.0 (3)
C10—C5—Ru	130.95 (16)	C28—C29—H29	119.5
C1—C6—H6A	109.5	C30—C29—H29	119.5
C1—C6—H6B	109.5	C31—C30—C29	120.1 (3)
H6A—C6—H6B	109.5	C31—C30—H30	120.0
C1—C6—H6C	109.5	C29—C30—H30	120.0
H6A—C6—H6C	109.5	C32—C31—C30	120.0 (3)
H6B—C6—H6C	109.5	C32—C31—H31	120.0
C2—C7—H7A	109.5	C30—C31—H31	120.0
C2—C7—H7B	109.5	C31—C32—C33	121.2 (3)
H7A—C7—H7B	109.5	C31—C32—H32	119.4
C2—C7—H7C	109.5	C33—C32—H32	119.4
H7A—C7—H7C	109.5	C28—C33—C32	119.2 (3)
H7B—C7—H7C	109.5	C28—C33—H33	120.4
C3—C8—H8A	109.5	C32—C33—H33	120.4
C3—C8—H8B	109.5	C35—C34—C39	119.6 (2)
H8A—C8—H8B	109.5	C35—C34—P2	117.25 (16)
C3—C8—H8C	109.5	C39—C34—P2	123.04 (17)
H8A—C8—H8C	109.5	C34—C35—C36	120.1 (2)
H8B—C8—H8C	109.5	C34—C35—H35	120.0
C4—C9—H9A	109.5	C36—C35—H35	120.0
C4—C9—H9B	109.5	C37—C36—C35	120.3 (2)
H9A—C9—H9B	109.5	C37—C36—H36	119.8
C4—C9—H9C	109.5	C35—C36—H36	119.8
H9A—C9—H9C	109.5	C38—C37—C36	119.5 (2)
H9B—C9—H9C	109.5	C38—C37—H37	120.2
C5—C10—H10A	109.5	C36—C37—H37	120.2
C5—C10—H10B	109.5	C37—C38—C39	121.0 (2)
H10A—C10—H10B	109.5	C37—C38—H38	119.5
C5—C10—H10C	109.5	C39—C38—H38	119.5
H10A—C10—H10C	109.5	C38—C39—C34	119.5 (2)
H10B—C10—H10C	109.5	C38—C39—H39	120.3
C16—C11—C12	118.64 (19)	C34—C39—H39	120.3
			
C3—Ru—P1—N1	−90.96 (9)	C2—C3—C4—C9	−167.3 (2)
C4—Ru—P1—N1	−101.90 (11)	C8—C3—C4—C9	−1.7 (3)
C5—Ru—P1—N1	−38.14 (16)	Ru—C3—C4—C9	126.6 (2)
C1—Ru—P1—N1	−25.85 (9)	C2—C3—C4—Ru	66.14 (13)
P2—Ru—P1—N1	157.33 (6)	C8—C3—C4—Ru	−128.2 (2)
C2—Ru—P1—N1	−54.59 (8)	C3—Ru—C4—C5	118.19 (18)
Cl1—Ru—P1—N1	65.77 (6)	C1—Ru—C4—C5	37.90 (12)
C3—Ru—P1—C17	23.72 (9)	P2—Ru—C4—C5	−122.04 (12)
C4—Ru—P1—C17	12.79 (12)	C2—Ru—C4—C5	79.24 (13)
C5—Ru—P1—C17	76.55 (16)	P1—Ru—C4—C5	135.82 (11)
C1—Ru—P1—C17	88.84 (10)	Cl1—Ru—C4—C5	−29.28 (14)
P2—Ru—P1—C17	−87.98 (7)	C5—Ru—C4—C3	−118.19 (18)
C2—Ru—P1—C17	60.10 (9)	C1—Ru—C4—C3	−80.29 (14)
Cl1—Ru—P1—C17	−179.54 (7)	P2—Ru—C4—C3	119.77 (12)
C3—Ru—P1—C11	145.64 (10)	C2—Ru—C4—C3	−38.95 (12)
C4—Ru—P1—C11	134.71 (12)	P1—Ru—C4—C3	17.63 (17)
C5—Ru—P1—C11	−161.54 (16)	Cl1—Ru—C4—C3	−147.47 (11)
C1—Ru—P1—C11	−149.24 (10)	C3—Ru—C4—C9	−118.1 (3)
P2—Ru—P1—C11	33.93 (8)	C5—Ru—C4—C9	123.8 (3)
C2—Ru—P1—C11	−177.99 (10)	C1—Ru—C4—C9	161.7 (3)
Cl1—Ru—P1—C11	−57.62 (8)	P2—Ru—C4—C9	1.7 (2)
C3—Ru—P2—C34	80.02 (10)	C2—Ru—C4—C9	−157.0 (3)
C4—Ru—P2—C34	45.70 (10)	P1—Ru—C4—C9	−100.4 (2)
C5—Ru—P2—C34	13.08 (11)	Cl1—Ru—C4—C9	94.5 (3)
C1—Ru—P2—C34	10.91 (14)	C3—C4—C5—C1	−1.4 (2)
C2—Ru—P2—C34	81.63 (13)	C9—C4—C5—C1	169.5 (2)
P1—Ru—P2—C34	−174.49 (8)	Ru—C4—C5—C1	−62.25 (14)
Cl1—Ru—P2—C34	−78.66 (8)	C3—C4—C5—C10	−171.1 (2)
C3—Ru—P2—C28	−159.27 (11)	C9—C4—C5—C10	−0.2 (4)
C4—Ru—P2—C28	166.41 (11)	Ru—C4—C5—C10	128.0 (2)
C5—Ru—P2—C28	133.79 (11)	C3—C4—C5—Ru	60.84 (14)
C1—Ru—P2—C28	131.61 (14)	C9—C4—C5—Ru	−128.2 (2)
C2—Ru—P2—C28	−157.67 (13)	C2—C1—C5—C4	−1.7 (2)
P1—Ru—P2—C28	−53.79 (10)	C6—C1—C5—C4	−176.0 (2)
Cl1—Ru—P2—C28	42.05 (10)	Ru—C1—C5—C4	62.28 (14)
C3—Ru—P2—Cl2	−34.56 (7)	C2—C1—C5—C10	168.8 (2)
C4—Ru—P2—Cl2	−68.88 (7)	C6—C1—C5—C10	−5.5 (3)
C5—Ru—P2—Cl2	−101.50 (7)	Ru—C1—C5—C10	−127.2 (2)
C1—Ru—P2—Cl2	−103.67 (11)	C2—C1—C5—Ru	−64.02 (14)
C2—Ru—P2—Cl2	−32.95 (10)	C6—C1—C5—Ru	121.7 (2)
P1—Ru—P2—Cl2	70.92 (3)	C3—Ru—C5—C4	−37.47 (12)
Cl1—Ru—P2—Cl2	166.76 (3)	C1—Ru—C5—C4	−117.22 (18)
C17—P1—N1—C23	−161.77 (15)	P2—Ru—C5—C4	64.67 (12)
C11—P1—N1—C23	93.12 (16)	C2—Ru—C5—C4	−80.07 (13)
Ru—P1—N1—C23	−40.00 (16)	P1—Ru—C5—C4	−98.89 (17)
C17—P1—N1—C27	62.39 (16)	Cl1—Ru—C5—C4	156.15 (12)
C11—P1—N1—C27	−42.73 (16)	C3—Ru—C5—C1	79.75 (13)
Ru—P1—N1—C27	−175.85 (12)	C4—Ru—C5—C1	117.22 (18)
C3—Ru—C1—C2	36.55 (12)	P2—Ru—C5—C1	−178.11 (11)
C4—Ru—C1—C2	79.42 (14)	C2—Ru—C5—C1	37.14 (12)
C5—Ru—C1—C2	116.33 (19)	P1—Ru—C5—C1	18.3 (2)
P2—Ru—C1—C2	119.67 (12)	Cl1—Ru—C5—C1	−86.63 (12)
P1—Ru—C1—C2	−54.36 (13)	C3—Ru—C5—C10	−163.2 (3)
Cl1—Ru—C1—C2	−150.20 (12)	C4—Ru—C5—C10	−125.7 (3)
C3—Ru—C1—C5	−79.78 (14)	C1—Ru—C5—C10	117.1 (3)
C4—Ru—C1—C5	−36.91 (13)	P2—Ru—C5—C10	−61.1 (3)
P2—Ru—C1—C5	3.34 (19)	C2—Ru—C5—C10	154.2 (3)
C2—Ru—C1—C5	−116.33 (19)	P1—Ru—C5—C10	135.4 (2)
P1—Ru—C1—C5	−170.69 (11)	Cl1—Ru—C5—C10	30.4 (2)
Cl1—Ru—C1—C5	93.46 (12)	N1—P1—C11—C16	92.25 (18)
C3—Ru—C1—C6	160.9 (2)	C17—P1—C11—C16	−11.1 (2)
C4—Ru—C1—C6	−156.3 (2)	Ru—P1—C11—C16	−137.91 (16)
C5—Ru—C1—C6	−119.4 (2)	N1—P1—C11—C12	−80.18 (16)
P2—Ru—C1—C6	−116.03 (19)	C17—P1—C11—C12	176.48 (15)
C2—Ru—C1—C6	124.3 (3)	Ru—P1—C11—C12	49.67 (17)
P1—Ru—C1—C6	69.9 (2)	C16—C11—C12—C13	1.4 (3)
Cl1—Ru—C1—C6	−25.90 (19)	P1—C11—C12—C13	174.28 (16)
C5—C1—C2—C3	4.2 (2)	C11—C12—C13—C14	−0.4 (3)
C6—C1—C2—C3	178.2 (2)	C12—C13—C14—C15	−0.6 (3)
Ru—C1—C2—C3	−58.77 (14)	C13—C14—C15—C16	0.6 (4)
C5—C1—C2—C7	−165.66 (19)	C14—C15—C16—C11	0.3 (4)
C6—C1—C2—C7	8.4 (3)	C12—C11—C16—C15	−1.3 (3)
Ru—C1—C2—C7	131.4 (2)	P1—C11—C16—C15	−173.59 (17)
C5—C1—C2—Ru	62.99 (13)	N1—P1—C17—C22	−162.32 (17)
C6—C1—C2—Ru	−123.0 (2)	C11—P1—C17—C22	−57.91 (18)
C3—Ru—C2—C1	−119.64 (18)	Ru—P1—C17—C22	73.97 (17)
C4—Ru—C2—C1	−80.35 (14)	N1—P1—C17—C18	21.93 (18)
C5—Ru—C2—C1	−38.50 (13)	C11—P1—C17—C18	126.33 (17)
P2—Ru—C2—C1	−122.13 (13)	Ru—P1—C17—C18	−101.79 (16)
P1—Ru—C2—C1	132.90 (12)	C22—C17—C18—C19	−1.3 (3)
Cl1—Ru—C2—C1	34.97 (14)	P1—C17—C18—C19	174.52 (17)
C4—Ru—C2—C3	39.29 (12)	C17—C18—C19—C20	0.3 (3)
C5—Ru—C2—C3	81.14 (13)	C18—C19—C20—C21	0.9 (4)
C1—Ru—C2—C3	119.64 (18)	C19—C20—C21—C22	−1.1 (4)
P2—Ru—C2—C3	−2.49 (17)	C20—C21—C22—C17	0.1 (4)
P1—Ru—C2—C3	−107.45 (11)	C18—C17—C22—C21	1.1 (3)
Cl1—Ru—C2—C3	154.61 (10)	P1—C17—C22—C21	−174.87 (18)
C3—Ru—C2—C7	120.4 (3)	C27—N1—C23—C24	−61.0 (2)
C4—Ru—C2—C7	159.7 (3)	P1—N1—C23—C24	160.01 (15)
C5—Ru—C2—C7	−158.4 (3)	N1—C23—C24—C25	56.8 (3)
C1—Ru—C2—C7	−119.9 (3)	C23—C24—C25—C26	−52.6 (3)
P2—Ru—C2—C7	117.9 (2)	C24—C25—C26—C27	52.8 (3)
P1—Ru—C2—C7	13.0 (2)	C23—N1—C27—C26	61.7 (2)
Cl1—Ru—C2—C7	−85.0 (2)	P1—N1—C27—C26	−159.53 (15)
C1—C2—C3—C4	−5.0 (2)	C25—C26—C27—N1	−57.6 (3)
C7—C2—C3—C4	164.60 (19)	C34—P2—C28—C33	−115.1 (2)
Ru—C2—C3—C4	−65.15 (14)	Cl2—P2—C28—C33	−15.7 (2)
C1—C2—C3—C8	−170.2 (2)	Ru—P2—C28—C33	115.29 (19)
C7—C2—C3—C8	−0.5 (3)	C34—P2—C28—C29	62.84 (19)
Ru—C2—C3—C8	129.7 (2)	Cl2—P2—C28—C29	162.24 (16)
C1—C2—C3—Ru	60.10 (14)	Ru—P2—C28—C29	−66.80 (19)
C7—C2—C3—Ru	−130.3 (2)	C33—C28—C29—C30	−0.6 (4)
C5—Ru—C3—C4	36.34 (13)	P2—C28—C29—C30	−178.6 (2)
C1—Ru—C3—C4	78.93 (14)	C28—C29—C30—C31	0.9 (4)
P2—Ru—C3—C4	−67.07 (13)	C29—C30—C31—C32	−0.6 (5)
C2—Ru—C3—C4	114.37 (18)	C30—C31—C32—C33	0.1 (5)
P1—Ru—C3—C4	−168.26 (12)	C29—C28—C33—C32	0.1 (3)
Cl1—Ru—C3—C4	65.05 (18)	P2—C28—C33—C32	177.94 (19)
C4—Ru—C3—C2	−114.37 (18)	C31—C32—C33—C28	0.2 (4)
C5—Ru—C3—C2	−78.03 (13)	C28—P2—C34—C35	−94.28 (19)
C1—Ru—C3—C2	−35.44 (11)	Cl2—P2—C34—C35	165.03 (17)
P2—Ru—C3—C2	178.56 (10)	Ru—P2—C34—C35	41.3 (2)
P1—Ru—C3—C2	77.37 (11)	C28—P2—C34—C39	81.0 (2)
Cl1—Ru—C3—C2	−49.32 (18)	Cl2—P2—C34—C39	−19.66 (19)
C4—Ru—C3—C8	120.2 (3)	Ru—P2—C34—C39	−143.34 (16)
C5—Ru—C3—C8	156.5 (2)	C39—C34—C35—C36	1.6 (4)
C1—Ru—C3—C8	−160.9 (2)	P2—C34—C35—C36	177.13 (19)
P2—Ru—C3—C8	53.1 (2)	C34—C35—C36—C37	0.0 (4)
C2—Ru—C3—C8	−125.4 (3)	C35—C36—C37—C38	−1.4 (4)
P1—Ru—C3—C8	−48.1 (2)	C36—C37—C38—C39	1.2 (4)
Cl1—Ru—C3—C8	−174.74 (16)	C37—C38—C39—C34	0.5 (4)
C2—C3—C4—C5	3.9 (2)	C35—C34—C39—C38	−1.9 (3)
C8—C3—C4—C5	169.6 (2)	P2—C34—C39—C38	−177.08 (18)
Ru—C3—C4—C5	−62.19 (15)		

### Hydrogen-bond geometry (Å, °)

**Table:**

*D*—H···*A*	*D*—H	H···*A*	*D*···*A*	*D*—H···*A*
C6—H6B···Cl1	0.98	2.71	3.426 (2)	131
C12—H12···Cl1	0.95	2.68	3.559 (2)	154
C18—H18···N1	0.95	2.56	2.984 (3)	107
C22—H22···Cl2	0.95	2.66	3.391 (2)	135
C23—H23A···Cl1	0.99	2.71	3.432 (2)	130
C29—H29···Cl1	0.95	2.78	3.414 (3)	125
C33—H33···Cl2	0.95	2.68	3.175 (3)	113
C37—H37···Cl1^i^	0.95	2.81	3.716 (2)	159
C39—H39···Cl2	0.95	2.56	3.088 (2)	115

Symmetry codes: (i) −*x*+1, *y*+1/2, −*z*+1/2.
